# Predictors of the utilisation of continuum of maternal health care services in India

**DOI:** 10.1186/s12913-022-07876-9

**Published:** 2022-05-05

**Authors:** Sumirtha Gandhi, Supriya Gandhi, Umakant Dash, M. Suresh Babu

**Affiliations:** 1 Bengaluru Dr. B.R. Ambedkar School of Economics, Bengaluru, Karnataka India; 2 Dayal Singh, College, Delhi University, New Delhi, India; 3 Institute of Rural Management, Anand, Gandhinagar, Gujarat India; 4 Indian Institute of Technology, Madras, India

**Keywords:** Continuum of maternal health care services, Demographic health survey, Multinomial logistic regression model, India

## Abstract

**Background:**

Utilisation of continuum of maternal health care services is crucial for a healthy pregnancy and childbirth and plays an important role in attaining Universal Health Coverage (UHC) and Sustainable Development Goals (SDGs) related to maternal and child health. This paper aims to assess the percentage of dropouts across various stages of utilization of continuum of maternal health services (CMHS) in India and also investigates the factors hindering the utilization of these services.

**Methods:**

We used recent data from National Family Health Survey(NFHS) encompassing a total sample of 1,70,937 pregnant women for the period 2015–16. The percentage of women dropping out while seeking maternal health care is measured using descriptive statistics. While, the factors impeding the utilization of maternal health services is estimated using a Multinomial Logistic Regression Model, where dependent variable (CMHS) is defined as complete care, incomplete care and no care.

**Results:**

Only17% of pregnant women availed the utilisation of complete care and 83% either did not seek any care or dropped after seeking one or two services. For instance, it is found that 79% of women who registered for antenatal care services (ANC) did not avail the same adequately. An empirical investigation of determinants of inadequate utilization of CMHS revealed that factors like individual characteristics, for instance- access to media (RRR: 2.06) and mother’s education play (RRR: 3.61) a vital role in the uptake of CMHS. It is also found that the interaction between wealth index and place of residence plays a pivotal role in seeking complete care. Lastly, the results revealed that male participation (RRR: 2.69) and contacting multi-purpose worker (MPW) (RRR: 2.33) are also at play.

**Conclusion:**

The study suggests that the major determinants of utilisation of CMHS are access to media, mother’s education, affordability barriers and male participation. Hence, policy recommendations should be oriented towards strengthening these dimensions and the utilisation of adequate ANC has to be considered as the need of the hour.

## Background

Utilisation of continuum of maternal health care services (CMHS) lays a strong foundation for healthy pregnancy and childbirth. It has been surmised that, pregnancy related complications arising during and after pregnancy obstructs maternal and child health in multiple ways, sometimes being fatal. The estimates so far (Sustainable Development Goals report, 2018) discern that around 293,760^1^ women in the world died due to pregnancy related complications in 2017. It was also found that these deaths were mainly compounded by numbers in developing nations with India being a major contributor. Moreover, 75% of India’s maternal deaths were associated with high blood pressure, severe bleeding and infections occurring during and after pregnancy [1]. However, these deaths can be prevented if adequate maternal care services are provided during the prenatal stage and, safe delivery systems and essential post-natal care services are in place [2, 3]. Together, these strategies form the cornerstone of CMHS. Considering the long and short term benefits of CMHS, the Government of India (GOI) implemented two important programmatic interventions namely, National Rural Health Mission (NRHM) in 2005–06^2^ and the Reproductive, Maternal, New-born, Child and Adolescent Health (RMNCH +A) programme in 2013 (RMNCH+A, 2013). But, there is a huge dearth of research in understanding the determinants of utilisation of CMHS and the dropouts across different stages of continuum of care is missing in the literature. In fact, the existing work is majorly oriented towards analysing the utilisation of individual maternal health care services in India [4–6]. The information would enable us to prescribe new policies/ revamp the existing ones to strengthen the maternal and child health programmes and ensure universal coverage of CMHS in India. Therefore, this study aims to investigate two main objectives. The first objective deals with the measurement of percentage of pregnant women dropping out across various stages of care seeking pathway and the second objective intends to analyse the determinants of complete care vs no care, complete care vs incomplete care and incomplete care vs no care.

### Conceptual framework

**Fig. 1:**
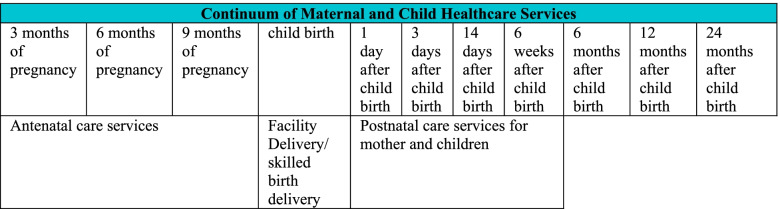
Framework of continuum of maternal health care services

CMHS relies on a valid presumption that the sequel of services provided throughout the lifecycle of pregnancy are interlinked. This is a recurrent theme in the Lancet Neonatal Survival Series and laid a strong premises in a conceptual framework formulated by Partnership of Maternal, New-born and Child health [7]. CMHS has two dimensions: the time dimension and the space dimension [7, 8]. According to the time dimension, a natural link exists between different time segments of pregnancy care, which spans from the early stages of pregnancy (antenatal care services) and childbirth (skilled-based delivery) to post-pregnancy stages (postnatal care services). The conceptualisation of these linkages are presented in Fig. 1. Meanwhile, the space dimension indicates “a seamless continuum of maternal health care services spanning from home to community, to the health centre and finally to the hospital” [7].

**Fig. 2:**
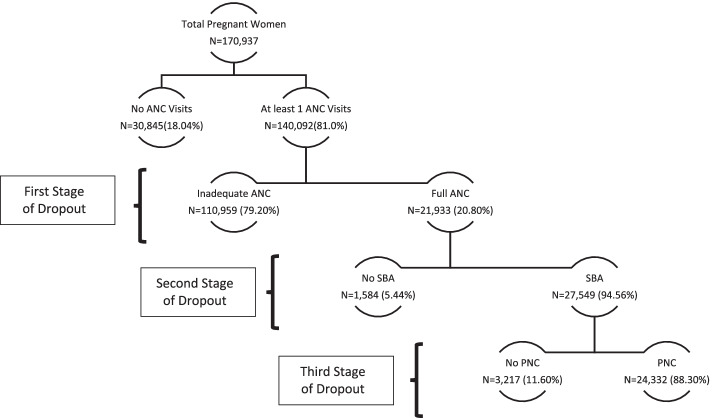
Conceptual framework of determinants of continuum of care

To examine the determinants of the uptake of continuum of maternal health care, existing conceptual frameworks [2] has been used to construct an overarching conceptual framework. This is demonstrated in Fig. 2. The framework encompasses determinants such as, the individual-level characteristics (age at marriage, education and parity), the household-level determinants (family size, caste, religion, income status), the community-level determinants (place of residence and region) and the policy level parameters (ASHA) – these determinants are hypothesised to influence the utilisation of CMHS and consequently improve the functioning and well-being of mother and her new-born.

## Data and methodology

### Data source

We have used the National Family Health Survey (NFHS) dataset, conducted in 2015–16^3^, spearheaded by the International Institute of Population Studies (IIPS). The target population of the survey were women aged between 15 and 49 years, who had at least one live birth in the previous 5 years. The survey entailed an independent sampling strategy for rural and urban areas. For the rural areas, a two-stage stratified sampling was employed wherein villages were used as the first stage unit (FSU) and the household as second. For the urban areas, a three-stage stratified sampling strategy was adopted, with the first stage being the ward, the second being the census enumeration block (CEB), and the third being the households. A total sample of this round constituted of 601,509 households and 699,686 individual women. However, for the current study, we restrict our analysis to women’s file (IR) which provides data on the maternal health utilisation, obtained by asking mothers about the number of antenatal visits, the consumption of iron-folic tablets, uptake of tetanus toxoid injections, the type of provider approached for child birth and whether the respondent’s health was checked immediately after delivering the baby. All the observations with missing values are dropped from the analysis and a final sample of 1,70,937 is utilized for the study.

### Description of variables

In this section, we provide a detailed description of dependent and independent variables of the model. We selected the variables based on the existing literature and availability of information in the dataset.

### Outcome variables

Although the utilisation of CMHS has been conceded as effective instrument for the increase of wellbeing of mother and child. However, in reality we found that the continuum of maternal health care services are not always consumed in sequence. In other words, most of the pregnant women have sought only two maternal health care services (ANC & SBA, SBA & PNC and ANC & PNC). Furthermore, some have availed only one maternal health care intervention (ANC/ SBA/PNC) in their entire lifecycle of pregnancy. Thus, the level of benefits certainly varies with the number of interventions availed.

Therefore, we disaggregate the utilisation of CMHS into three possible categories - no care, partial care and full care. First, we calculated the utilisation of adequate ANC, SBA and PNC services. ‘Adequate ANC’ is defined as women having four or more ANC visits, consumed 100 iron and folic acid tablets/ syrup for their last birth and availed at least two tetanus injections. A women had ‘adequate SBA’ if her last delivery was attended by a qualified personnel such as a doctor, nurse, auxiliary midwife, clinical officer or trained birth attendant. The utilisation of ‘PNC’ was considered adequate if she had undertaken a health check-up at any health facility within 2 days of delivery for their last birth. Using these three indicators, we constructed our dependent variable as a categorical variable denoted as ‘0’ (No Care) if none of these services were undertaken, ‘1’ (Partial Care) if any one or two of the aforementioned services were availed and ‘2’ (Full Care) if all three services were utilised.

### Explanatory variables

The explanatory variables considered in this analysis are divided into five groups- individual-level, household-level, community-level, access related barriers and policy level characteristics.

### Statistical analysis

Since, the dependent variable of this study is a polychotomous variable with three categories, a Multinomial Logistic Regression Model (MNLM) introduced by Maddala [9] was deemed most appropriate.. Nevertheless, in case of a categorical dependent variable, a researcher is confused to choose either MNLM or Ordered Logistic Regression Model (OLRM). The distinguishing feature delineating these two models is the very assumption of *Parallel Slopes*, according to which, a given explanatory variable affects the likelihood of an individual being in particular category in the same way as it affects the likelihood of the same individual falling in another category. Borooah [10] explains that, if the assumption is violated, then the MNLM methodology is considered more appropriate compared to the OLRM. Furthermore, when ordered nature of the variable is not very obvious, then it is recommended to treat it as a non-ordered variable and hence, a MNLM is recommended. This is because, when a non-ordered (ordered) variable is treated as an ordered (non-ordered) it results in issues like resulting in biased estimation, and model misspecification for the former case and efficiency loss for the latter. Under these circumstances, the loss of efficiency is a less serious problem compared to the biased estimation. Since, the categories of our dependent variable do not imply any ordering, hence we treat our dependent variable as non-ordered and use MNLM for the empirical analysis. To check the suitability of the model, we conducted diagnostic tests like, likelihood ratio test and the Small-Hsiao test [11]. All the empirical analysis were conducted using the statistical software STATA, version (15.0).

To explore the relationship between the different sets of covariates and utilization of CMHS, we estimated two models. In the first model, we considered e ‘No care’ as the base outcome, while in the second model, ‘Partial care’ was considered as the base outcome. We discuss our findings across 1) Full care vs No care, 2) Incomplete care vs No care and 3) Full care vs Incomplete care. This helps us glean valuable and new insights previously unexplored in CMHS studies [7, 8]. In order to estimate the determinants of the continuum of maternal health care services, we specify the empirical model as follows:

1 $${O}_{ij}=\alpha +{\beta}_1{Individual}_i+{\beta}_2{Household}_i+{\beta}_3{Policy}_i+{\beta}_4{Community}_i+{\beta}_5{Barriers}_i+{\varepsilon}_i$$

where *O*_*ij*_ is a polychotomous variable depicting *i*^*t*h^ individual’s utilisation of ‘j’ health care services, *O*_*ij*_ equals 0,1, or 2 if the individual’s utilisation of maternal health care services is No-Care (No ANC, No SBA and No PNC), Partial/ Incomplete Care (Utilisation of either one or two services out of ANC, SBA and PNC) and Full Care (Availing all three services - ANC, SBA and PNC), respectively.

## Results

This section is divided into two segments; in the first segment, we understand the percentage of drop-outs across different stages of CMHS, and in the second segment, estimated results of the MNLM is elucidated.

### Descriptive statistics

Table 1 shows that the percentage of women seeking complete care was only 16.82%, whereas around 73% sought partial care, and 10.6% of the respondents did not utilise any care. Around 56.05% of pregnant women married in the age group of 18–24, while 37.24% married before attaining the legal age of marriage. Nearly 70% of the women had 1–2 children. The majority (67.34%) of women had access to at least one medium of information. Among all the pregnant women, 46.58% availed of secondary education, around 28% were illiterate. The percentage of women with higher education was only 12.03%. The mean household size of a family was 6.4. Around 23.82% of pregnant women belonged to the poorest quintile, followed by poor (21.27%), middle (19.96%), rich (18.83%) and richest quintile population (16.12%), respectively. Caste-wise differentials demonstrated that most women belonged to Other Backward Caste (46.02%). The percentage of women belonging to the Hindu religion was 81%. Only 44% of Indian women had control over the household’s financial decisions. Region-wise disaggregation discerned that around 54% of women were from high-focussed states and 43% hailed from non-high focussed grouped states. Only 3.16% of the sample population belonged to north-eastern states. The percentage of women residing in rural areas was 71.60%, while only 28.40% lived in urban areas. During the first ANC visits, only 41% of pregnant women contacted an *Anganwadi* worker or Accredited Social Health Activist (ASHA).

**Table 1:** Summary statistics

Variables	2015–16 [N]	2015–16 [%]
**Dependent Variable**		
**Continuum of Care**		
Complete Care	28,744	16.82
Incomplete Care	124,072	72.58
No Care	18,120	10.60
**Individual-Level Characteristics**		
**Age at Marriage**		
Less than 18 years	63,656	37.24
18–24	95,808	56.05
25–49	11,472	6.71
**Access to Media**		
No Media	55,829	32.66
At least 1 Media	115,107	67.34
**Parity**		
No children	1711	1
1–2	119,839	70.11
3–4	39,639	23.19
5 or more	9745	5.70
**Education**		
Illiterate	47,793	27.96
Primary	22,957	13.43
Secondary	79,628	46.58
Higher	20,557	12.03
**Household-Level Characteristics**		
**Household Size**	6.4	
**Wealth Index**		
Poorest	40,713	23.82
Poor	36,361	21.27
Middle	34,120	19.96
Rich	32,194	18.83
Richest	27,547	16.12
**Caste**		
Scheduled Caste	37,870	22.15
Scheduled Tribe	18,308	10.71
Other Backward Caste	78,672	46.02
Others	36,086	21.11
**Religion**		
Hindu	138,346	80.93
Muslim	23,822	13.94
Christian	3534	2.07
Other	5232	3.06
**Proportion of Under 5 Children**		
Less than 2 under five children	151,586	88.68
More than 2 under five children	19,350	11.32
**Financial Autonomy**		
No	3270	11.03
Some	8356	44.60
High	7993	44.37
**Community-Level Characteristics**		
**Region**		
Non-High Focussed States	73,535	43.02
High North-East	5398	3.16
High -Focussed States	92,002	53.82
**Residence**		
Rural	122,398	71.60
Urban	48,538	28.40
**Community Poverty**		
Low	78,361	45.84
High	92,575	54.16
**Community Education**		
Low	87,947	51.45
High	82,989	48.55
**Contacting Community Health Worker**		
No	97,980	57.32
*Anganwadi* and ASHA	70,797	41.42
MPW	2160	1.26
**Access-related barriers**		
Acceptability	7122	5.87
Affordability	4345	3.58)
Availability	109,798	90.54
**Total**	**1,70,937**	

Source: Author’s Computation

#### Weakest and strongest link in the CMHS pathway

**Fig. 3:**
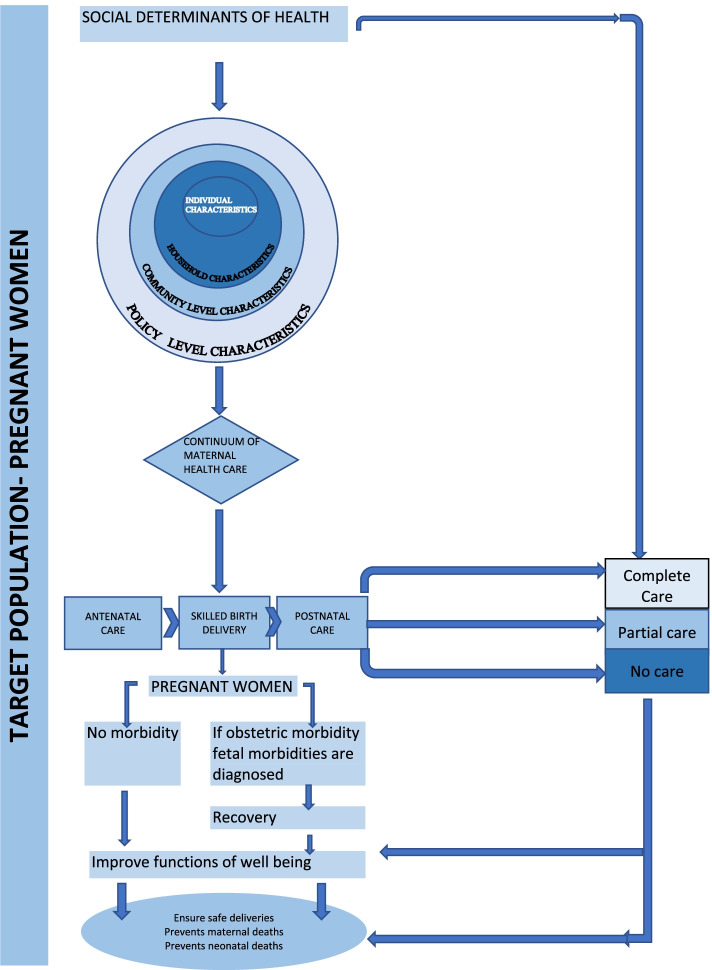
Various stages of dropouts in the utilisation of CMHS

Figure 3 illustrates the entire mechanism of the care-seeking pathway. We tried to capture the sequential pattern of maternal healthcare- seeking behaviour by underpinning different stages of dropouts along with the determinants of CMHS.

Astonishingly, around 80% of the pregnant women availed at least one or more ANC visits, but only 20.80% of them sought adequate ANC services. The remaining 79% dropped out without availing adequate ANC services, thereby making it the *weakest link in the continuum of care pathway*. Of the adequate ANC users, most of the pregnant women availed SBA services (95%), making it the *most vital link to the CMHS pathway*. Among those who utilised adequate ANC and SBA, around 88.3% sought PNC care. We also noticed that the percentage of women dropping in the third stage was around 11.6%.

#### Drop-outs in the continuum of care pathway: across states

**Fig. 4:**
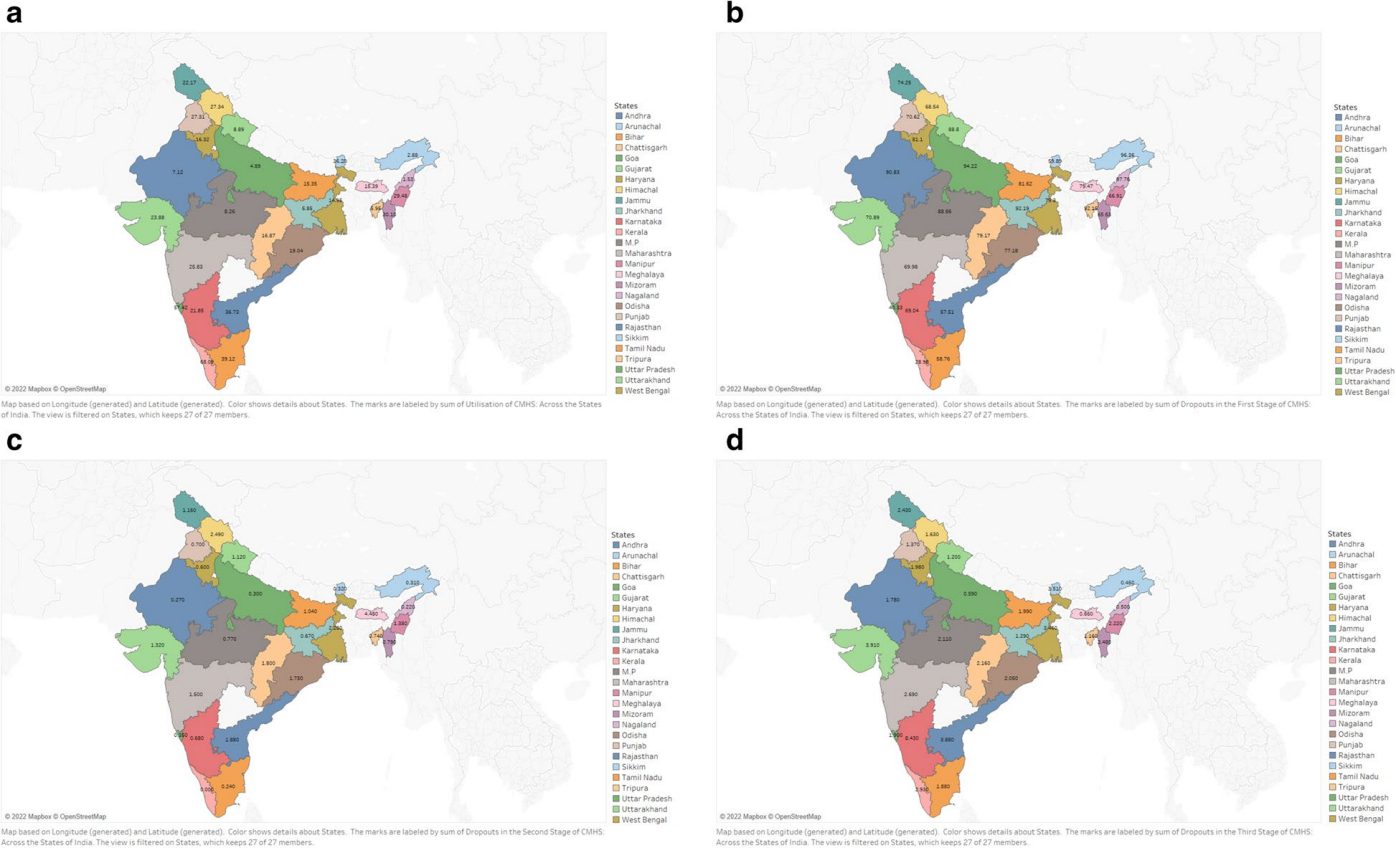
**a** Utilisation of CMHS: Across the States of India. **b** Dropouts in the First Stage of CMHS: Across the States of India. **c** Dropouts in the Second Stage of CMHS: Across the States of India. **d** Dropouts in the Third Stage of CMHS: Across the States of India

Figure 4a-d depicts the state-wise estimation of the utilisation of CMHS and dropouts at the first, second and third stages of the care seeking pathway. Figure 4a indicates that CMHS utilisation was highest in Kerala (68.09), followed by other southern states. States belonging to western ghats performed better than other states. Worst performance was recorded by the states belonging to Gangetic plains and Himalayan terrain.

The first stage of dropout indicates those women who registered for ANC in the first trimester but did not avail of adequate ANC services in India. State-wise variations are elucidated in Fig. 4b. We found the differences across states to be quite stark. For instance, in Nagaland, around 98% of women dropped out from the first stage of CMHS. Uttar Pradesh, Bihar, Jharkhand, Rajasthan, Uttarakhand and Madhya Pradesh demonstrate a worrying pattern. Similarly, the other northern and northeastern states witnessed very high dropout rates in the first stage of CMHS. The state of Kerala (29%) recorded the lowest dropout rates, the number being high at the same time.

The Second stage of dropout (Fig. 4c) indicates the percentage of women availing ANC services but forgoing SBA is relatively lower, making it the strongest linkage in the continuum of care pathway. State-wise estimation presented below indicates negligible variations.

Figure 4d depicts the third stage of dropout across the states of India. The proportion of women who undertook ANC and SBA but did not avail PNC. It ranges between 0.45 and 8.43%. It was also found that the dropout rates in this stage are comparatively higher in southern parts of India. The lowest dropout rates were witnessed in the North-eastern region.

#### Predictors of CMHS in India

Empirical estimation based on the Multinomial logistic regression model (MNLM) propounded by Mc Fadden (1984) revealed that a myriad of factors determines CMHS utilisation. We disaggregated these factors into household level, individual-level, community level, health-related factors and access-related barriers. We estimated two separate models to gather maximum insights on all possible alternatives. In the first model, no care was used as the base outcome, while in the second, incomplete care was chosen as the base outcome. The results of MNLM are presented in Table 2. We have used the terms - complete care/ full care and partial care/incomplete care interchangeably.

**Table 2:** Determinants of utilisation of CMHS in India, 2015–16

Independent Variable	Full Care Vs. No Care			Incomplete Care Vs. No Care			Complete Care Vs. Incomplete Care		
***Individual Level Characteristics***									
***Age at Marriage (Ref < 18)***									
	**RRR**	**95% CI**		**RRR**	**95% CI**		**RRR**	**95% CI**	
18–24	1.34* [0.04]	1.25	1.43	1.13* [0.03]	1.07	1.19	1.18*[0.03]	1.13	1.23
25–49	1.82* [0.15]	1.55	2.13	1.26* [0.09]	1.09	1.45	1.45*[0.05]	1.35	1.56
***Parity (Ref: < 1)***									
1 to 2	1.24 [0.18]	0.93	1.65	1.18 [0.14]	0.94	1.48	1.05 [0.10]	0.87	1.28
3 to 5	0.70**[0.11]	0.52	0.94	0.84 [0.10]	0.66	1.05	0.84***[0.09]	0.69	1.02
5 or more	0.44* [0.08]	0.32	0.62	0.81***[0.10]	0.64	1.03	0.55* [0.07]	0.42	0.7
***Education (Ref: Illiterate)***									
Primary	1.21* [0.06]	1.1	1.33	1.10** [0.04]	1.03	1.18	1.10** [0.04]	1.02	1.18
Secondary	1.78* [0.08]	1.62	1.95	1.46* [0.05]	1.36	1.56	1.22* [0.04]	1.15	1.3
Higher	3.61* [0.37]	2.95	4.41	2.33* [0.22]	1.93	2.81	1.55* [0.06]	1.43	1.68
***Financial Autonomy (Ref: No)***									
Moderate	1.14 [0.14]	0.9	1.45	1.03 [0.10]	0.85	1.26	1.11 [0.09]	0.95	1.29
Full autonomy	1.30** [0.16]	1.01	1.66	0.97 [0.10]	0.79	1.18	1.34* [0.10]	1.15	1.57
Constant	1.03 [0.19]	0.72	1.48	7.48* [1.12]	5.58	10.03	0.15* [0.04]	0.08	0.27
***Access to Media (Ref: No)***									
At least 1 media	2.06* [0.09]	1.9	2.24	1.36* [0.04]	1.28	1.45	1.51* [0.05]	1.42	1.6
Household Size	0.94* [0.01]	0.93	0.95	0.97* [0.00]	0.97	0.98	0.97* [0.00]	0.96	0.98
***Household Level Characteristics***									
***Interaction of Wealth Index & Residence (Ref: Poorest Urban)***									
Poor Urban	1.42* [0.14]	1.18	1.71	1.31* [0.11]	1.11	1.55	1.08 [0.05]	0.98	1.19
Middle Urban	1.96* [0.22]	1.57	2.45	1.55* [0.16]	1.26	1.9	1.27* [0.06]	1.15	1.4
Rich Urban	2.66* [0.36]	2.04	3.48	1.95*[0.25]	1.51	2.51	1.37* [0.07]	1.23	1.51
Richest Urban	7.70* [1.98]	4.65	12.73	5.23* [1.32]	3.19	8.58	1.47* [0.08]	1.32	1.65
Poorest Rural	0.78* [0.06]	0.67	0.9	0.95 [0.06]	0.85	1.07	0.82* [0.05]	0.73	0.91
Poor Rural	0.99 [0.07]	0.86	1.15	1.13** [0.07]	1.01	1.27	0.88**[0.04]	0.8	0.96
Middle Rural	1.39* [0.10]	1.21	1.6	1.37* [0.08]	1.22	1.54	1.02 [0.04]	0.93	1.11
Rich Rural	1.75* [0.13]	1.51	2.03	1.62* [0.11]	1.42	1.84	1.08***[0.05]	1	1.18
Richest Rural	2.52* [0.22]	2.11	2.99	2.06* [0.17]	1.76	2.42	1.22* [0.05]	1.12	1.33
***Caste (Ref: SC)***									
ST	1.15** [0.06]	1.04	1.28	1.02 [0.04]	0.92	1.09	1.15* [0.04]	1.08	1.24
OBC	1.24* [0.05]	1.14	1.34	1.18* [0.04]	1.11	1.27	1.05***[0.03]	1	1.1
Others	1.14** [0.06]	1.03	1.26	1.12** [0.05]	1.02	1.23	1.02 [0.03]	0.96	1.07
***Religion (Ref: Hindu)***									
Muslim	0.52* [0.02]	0.47	0.57	0.62* [0.02]	0.58	0.67	0.83* [0.03]	0.78	0.88
Christian	0.93 [0.10]	0.74	1.15	0.70* [0.07]	0.57	0.85	1.33* [0.08]	1.18	1.5
Other	0.77** [0.08]	0.62	0.96	0.94 [0.09]	0.77	1.14	0.82* [0.04]	0.75	0.91
***Father Present during ANC visits (Ref: No)***									
Yes	2.33* [0.09]	2.16	2.51	1.52* [0.04]	1.44	1.61	1.53* [0.04]	1.45	1.62
***Community Level Characteristics***									
***State Group (Ref: Non-High Focussed)***									
North East	0.28* [0.02]	0.24	0.33	0.36* [0.03]	0.32	0.42	0.77* [0.04]	0.7	0.86
Major High Focused States	0.19* [0.01]	0.17	0.21	0.48* [0.02]	0.45	0.52	0.39* [0.01]	0.38	0.41
***Community Poverty Status (Ref: Low)***									
High	0.61* [0.03]	0.56	0.67	0.74* [0.03]	0.68	0.8	0.83* [0.02]	0.79	0.87
***Community Education Status (Ref: Low)***									
High	1.66* [0.07]	1.53	1.8	1.33* [0.05]	1.24	1.43	1.24* [0.03]	1.18	1.31
***Health Related Characteristics***									
***Barriers (Ref: Acceptability)***									
Affordability	1.02 [0.11]	0.82	1.26	1.11 [0.11]	0.92	1.35	0.91***[0.05]	0.82	1.01
Availability	0.82* [0.06]	0.71	0.94	0.99 [0.06]	0.87	1.12	0.83* [0.03]	0.77	0.88
***ANC Contact (None)***									
*Anganwadi* & ASHA	1.66* [0.05]	1.56	1.76	1.40* [0.04]	1.33	1.48	1.18* [0.02]	1.14	1.23
MPW	2.69* [0.38]	2.05	3.54	1.51*[0.19]	1.17	1.93	1.79* [0.12]	1.57	2.03

*Note*: *p < 0.001, **p < 0.05, ***p < 0.1. [] is the standard error

Source: Author’s Compilation

We found that women with higher education levels as compared to their illiterate counterparts have a higher likelihood to seek complete care over no care (RRR: 3.61, CI: 2.95–4.41), incomplete care over no care (RRR: 2.33, CI: 1.93–2.81) and complete care over incomplete care (RRR: 1.55, CI: 1.43–1.68). Women having access to at least one medium of information are more likely to gravitate towards complete care over no care (RRR: 2.06, CI: 1.90–2.24) and complete care over incomplete care (RRR: 1.51, CI: 1.42–1.60). Furthermore, in contrast to women of age less than 18 years at the time of marriage, women whose age was more than 18 years are more likely to seek complete maternal healthcare services over no/partial care category.

The impact of an interaction between wealth index and residence discerned that the relative risk ratio (RRR) of complete care over no care (RRR: 7.70, CI: 4.65–12.73), complete over incomplete care (RRR: 1.47, CI: 1.32–1.65) and incomplete care over no care (RRR: 5.23, CI: 3.19–8.58) is highest among the richest quintile women residing in urban areas as compared to the poorest ones. Compared to the poorest women residing in urban areas, the richest from rural areas is more likely to avail maternal health care across all three categories- highlighting the relevance of financial status of women in determining the utilisation of maternal health care services. Financial autonomy is also associated with a greater likelihood to receive complete care over incomplete care (RRR: 1.34, RRR: 1.15–1.57) and full care over no care (RRR: 1.03, CI: 1.01–1.66).

With an increase in the household size by one member, the RRR of utilising complete care over no care (RRR: 0.94, CI: 0.93–0.95), complete care over incomplete care (RRR: 0.97, CI: 0.96–0.98) and incomplete care over no care (RRR: 0.97, CI: 0.96–0.98) reduces. For all the three categories, a woman having contact with community health workers like ASHA, *Anganwadi* or Multipurpose Health Worker (MPW) has a higher RRR than those who did not contact CHWs. Similarly, participation of husband in care-seeking procedure increases the likelihood to seek complete care over no care (RRR: 2.33, CI: 2.16–2.51), incomplete care over no care (RRR: 1.52, CI: 1.44–1.61) and complete care over incomplete care (RRR: 1.53, CI: 1.45–1.62).

We found that a woman belonging to ST communities is more likely to go for complete care over no care (RRR: 1.15, CI: 1.04–1.28), complete care over incomplete care (RRR: 1.15, CI: 1.08–1.24) and incomplete care over no care (RRR: 1.02, CI: 0.92–1.09). Similarly, a woman belonging to the OBC community is more likely to seek complete care over no care, incomplete care over no care and complete care over incomplete care. It is found that Muslim women have a lower odds of utilising CMHS vis-à-vis the Hindu women.

A woman hailing from northeastern states and major high-focussed grouped states were found to have a lower odds of utilising complete care over no care, incomplete care over no care and complete care over incomplete care. Finally, an increase in the community’s education status is associated with a higher odds of seeking complete care over no care, complete care over incomplete care and incomplete care over no care. Affordability and availability barriers were found to reduce the likelihood to seek complete care over no care. It was also found that compared to acceptability barriers, the prevalence of affordability and availability issues are more responsible for drop-outs in the utilisation of CMHS. Finally, the influence of MPW was significant for the utilisation of complete care over no care and also for utilising complete care over incomplete care.

## Discussion

This study encapsulates the magnitude of dropouts across the continuum of care pathway and examines the effects of individual, household, community level characteristics, health-related characteristics and access-related barriers on the utilisation of CMHS in India for 2015–16. The major findings was that utilisation of continuum of maternal health care services was suboptimal and state-level variations were tremendous. The significant contributors of CMHS are mother’s education, access to media, male participation and contacting MPW.

The coverage of full care in India is found to be 16.82%, much lower than other developing countries [12, 13]. Around 72% of women availed partial care services indicating higher levels of dropouts in the continuum of care seeking pathway. An enquiry into the proportion of women dropping out at different stages of CMHS illuminated massive dropouts at the first stage of CMHS. Although 80% of women took one or more ANC visits, only 20.08% of them sought adequate ANC services, thereby making it the weakest link in the continuum of care pathway. This pattern has been explained by two possible reasons in the literature – first, the utilisation of adequate ANC services entails multiple visits to the facilities demanding the time spent on domestic chores and second, although the health care services are provided at free of cost at the government centres but indirect medical costs such as transportation, drugs are mostly incurred by out-of-pocket expenditure in India [14, 15].

We found that enormous amount of dropouts in the first stage of CMHS was majorly compounded by the low performing states (90%)- such as Bihar, Uttar Pradesh, Jharkhand and Rajasthan. While, the state of Kerala experienced lowest levels dropouts in the first stage of CMHS (29%). Previous studies have reported the interstate variation in the utilisation is mainly attributed to lack of education, availability and accessibility of maternal health care services [16]. The utilisation of maternal health care services are relatively higher in the southern states of India- Kerala, Tamil Nadu, Andhra Pradesh, Telangana and Karnataka.

The study’s finding revealed that the interaction of wealth index and place of residence is one of the significant determinant of CMHS. It is hypothesised that women belonging to higher income quintile receive better healthcare services because they mostly reside in urban areas embedded with better access to healthcare services. Nevertheless, we found that the impact of income is higher compared to the place of residence. The possible justification might be associated with the fact that women hailing from higher economic background (irrespective to their place of residence) are in better position to incur expenses on transportation to avail healthcare services. Similarly, the other studies conducted in low income countries found higher utilisation of maternal health care services among urban and wealthier women [17, 18].

The relationship between household size and utilisation of CMHS is well established in the literature. However, there is no clear consensus on the polarity of the impact as some argue that an increase in family size ushers greater support, increased concern and commitment towards pregnant women’s health and thereby increases the utilisation of maternal health care services. In contrast, some argue that an increase in family size limits the allocation of resources for pregnant women [19, 20]. Our results are in support of the latter argument.

Women who had lower education status were less likely to avail complete care than those who attain higher education. Consistently, the finding of other studies [13, 21–25] revealed a positive association of utilisation of maternal health care services with their educational status. This might be due to the fact that “highly educated women are more likely to be knowledgeable about their rights and health, have more exposure to the information of available health care services and have more self- confidence than those with non or less education [26].

Similar to the findings of previous studies conducted in different contexts [13, 20, 23, 24], the access to media of information was more likely to increase the utilisation of complete care over no care, incomplete care over no care and complete care over incomplete care. Moreover, in consistency with other related studies [13, 20, 23, 24], women with access to medium of information were more likely to have knowledge about the relevance and availability of healthcare services.

The significant positive association of women’s utilisation with male involvement is encouraging as it suggests increasing women’s participation in care seeking behaviour works synergistically with male participation. The male involved is positively associated with women empowerment. The involvement of spouses provides emotional support, physical assistance such as purchasing essential drugs and collective diagnostic reports [27, 28]. Our results also convey that, women who had a say in decision making process were more likely to be accompanied to seek maternal health care services by their male counterparts. It could be possibly attributed to higher levels of negotiating power among empowered women. Involvement of spouses may enhance women’s decision making power in other realms of social and economic spheres [29, 30].

To our knowledge, this study is the first to-date to examine the relationship between availability/affordability barriers and women’s decision to seek complete care. We constructed a composite measure to capture availability, affordability and acceptability barriers. Most studies examined single barriers. However, this analysis provides a composite scores to inform the association between various barriers and continuum of maternal health care services. Key findings indicated that affordability barrier was positively and significantly associated with increasing odds of utilising complete care with reference to no care and incomplete care. It is important to understand that, availability of a particular services is prerequisite for initial visits but financial status impacts the subsequent visits.

As hypothesised, the regional variable plays a crucial role in determining the utilisation of CMHS. We found that a woman residing in non-high focus group states is less likely to avail CMHS compared to the one hailing from major high focus group states and north-eastern region. This could be attributed to higher levels of political commitment towards social sector, robust health system and strong socio-economic status of women prevalent in non-high focus states [16]. In contrast to this, states belonging to major high focussed group are characterised by limited health infrastructure, lack of political commitment and poor socio-economic status. Jeffery and Jeffery (2010) [31] indicate that these regions have systematic discrepancies like lack of equipment, medicines, blood, corruption and discriminatory treatment towards different sub groups of populations.

Finally, this study elucidates the importance of health workers in strengthening the CMHS pathways. Woman who met MPW in her initial visits were more likely to seek continuum of maternal health care services compared to those who did not meet MPW in their initial visits. The involvement of MPW would strengthen women’s knowledge about the relevance of continuum of care and complications associated with incomplete or no care [32]. Such efforts may be most impactful in settings where women’s knowledge about availability of health care services are lowest, as was the case in India.

## Conclusion

This study makes a major contribution in the area of maternal and child health care. Disaggregation of CMHS into three important categories like complete, incomplete and no care are pertinent to suggest micro-level policies to promote CMHS. Findings of this study highlight that, a significant proportion of women drops out without availing all the essential health care services in the continuum of care pathway, indicating a higher proportion of women utilising partial care. The dropouts across each stage of care indicated that weakest link is the first stage of CMHS, and second stage of CMHS turned out to be a strongest link. Estimation at the state-level indicates that even the best performing states such as Kerala experience a dropout of around 29% in this first stage of CMHS. Moreover, the condition of low-performing states of India is even more debilitating with each of them indicating a dropout of around 90% in this stage. The empirical findings of this study suggested that impact of income and affordability barriers are relatively higher in explaining the utilisation of partial care to complete care. Other factors increasing the likelihood to avail complete care are male participation, educational status, access to media and contacting MPW.

This paper brings forth certain policy recommendations crucial to boost the utilisation of complete maternal health care services. In doing so, greater efforts should be undertaken to strengthen the weakest linkage of CMHS, which would in turn increase the utilisation of CMHS. First step towards this could be the states considering that an increase the utilisation of adequate ANC is the need of the hour. Since, our empirical findings indicate that the impact of income and affordability barriers is more pronounced in explaining the utilisation of partial care relative to full care. Hence, government might provide cash-based incentives to increase and ensure adequate utilisation of ANC services in India and at state-level. As a medium run strategy, awareness pertaining to the benefits of CMHS should be disseminated to both women and their husbands. This could be done by strengthening mobile phone communication strategies, sending texts and audio messages in vernaculars, newspapers, radio and television and conducting community level awareness programs. Ensuring utilisation of CMHS will require strengthening the role of community health workers also. This is because they act as direct interface between pregnant women and the health system. Furthermore, they can be used as a catalysts in tracking women during the entire lifecycle of pregnancy and following them up if appointments are missed. Hence, government should concert efforts in increasing their wages competitively, providing proper reimbursement schemes for out-of-pocket expenses and streamlining the payment process related to health works.

### Limitations and strengths of the study

The study provides an evidence of social determinants of health that motivate pregnant women to undertake the continuum of maternal health care services. However, these findings need be interpreted with some caution. Some of the limitations of this study are:

The continuum of maternal health care services and dropouts across each levels of pregnancy would be determined by demand and supply-side factors, we could not include important supply-side covariates like patient-provider relationship, availability of workforce, etc. in our model due to the unavailability of data. Also, factors related to inertia/ status quo bias could not be included due to data constraints. Although the space dimension plays an important role in strengthening different levels of health care services, we were unable to assess the role of this dimension with the available information. Therefore, the study’s findings may provide not provide the complete list of indicators contributing to the dropouts and incomplete utilisation of care. Moreover, our results cannot be generalised to all women of reproductive age because the survey design is restricted to married and cohabiting women only. Hence, a qualitative research adopting a mixed approach would help to elucidate a comprehensive list of factors contributing to the dropouts/ incomplete utilisation of continuum of maternal health care services.

## Data Availability

The dataset analysed during the current study are available in the DHS Program website, https://dhsprogram.com/data/available-datasets.cfm

## Footnotes

1 Further information is available at: https://ourworldindata.org/maternal-mortality

2 Renamed as National Health Mission (NHM) in 2013.

3 Dataset can be obtained from https://dhsprogram.com/data/dataset_admin/login_main.cfm
